# Two cases of perforated corneal ulcers complicating rheumatoid arthritis treated successfully by biological therapy

**DOI:** 10.1186/s41927-019-0108-1

**Published:** 2020-02-07

**Authors:** Sara Benchérifa, Bouchra Amine, Imane El Binoune, Samira Rostom, Rachid Bahiri

**Affiliations:** Department of Rheumatology A, El Ayachi Hospital-Salé, CHU Ibn Sina, University Mohammed V-, Rabat, Morocco

**Keywords:** Perforated corneal ulcer, Biologic agents, Stabilization

## Abstract

**Background:**

Peripheral ulcerative keratitis (PUK) is a severe inflammatory ocular disease that can affect patients with a long history of rheumatoid arthritis (RA). The use of biotherapy has revolutionized the treatment of the RA and has provided encouraging outcomes especially in the treatment of PUK reported in few cases. In this article, we describe the case of two patients with the history of perforated corneal ulcer complicating RA treated successfully by biologic agents.

**Case presentation:**

**Case 1:** A 45-year-old woman was diagnosed for over 17 years with sero-positive RA refractory to conventional synthetic disease-modifying antirheumatic drugs (csDMARDs). She had received one cycle of Rituximab with clinical and biological failure. In July 2017, she presented an active RA flare with a painful left eye and a decreased visual acuity. Ocular examination revealed a corneal perforation in the left eye and a pre-perforation in the right eye. She received an emergency bolus of methylprednisolone 1 g/day during three consecutive days and was followed by Infliximab. After thirteen months, Infliximab was effective on the rheumatic disease and on the corneal involvement as it stopped its gradual perforation in the right eye, and stabilized corneal ulcer in the left eye.

**Case 2:** A 68-year-old man had been diagnosed since 2010 with sero-positive RA refractory to csDMARDs complicated in July 2017 by corneal perforation in the right eye. He was hospitalized for his ocular involvement and his active RA. He received an emergency bolus of methylprednisolone 500 mg/day during three consecutive days and was followed by Rituximab. After six months, we observed the stabilization of the right eye corneal damage and the resolution of articular symptoms.

**Conclusions:**

Our cases suggest the efficacy of Infliximab (case 1) and Rituximab (case 2) as a treatment of this severe and destructive keratolysis of the cornea complicating an active RA allowing to plan corneal graft. This positive therapeutic response will contribute to increase literature reports of this therapy success.

## Background

Rheumatoid arthritis (RA) is a chronic systemic inflammatory disease involving primarily the synovium of joints but can affect other organs including the eye. The ocular manifestations that can occur during the course of RA or that can be the initial sign of the disease are multiple and can include dry-eye syndrome, episcleritis, scleritis, sclero-uveitis, retinal vasculitis and peripheral ulcerative keratitis [1, 2]. Physicians can misdiagnose that ocular involvement. Therefore, rheumatologists should perform an ocular examination for all RA patients during the diagnosis and the follow-up. PUK and necrotizing scleritis are the two most severe ocular manifestations associated with the RA. Untreated, they are sight threatening with high mortality rate due to their association with systemic vasculitis [3, 4]. Therefore, early diagnosis and treatment are recommended in collaboration with the ophthalmologists [3]. Recently, PUK in patients with RA has become less common due to improved treatment of RA particularly with biological therapies [1]. Few publications reported the effectiveness of the biologic agents as a treatment of PUK related to RA. Meanwhile, our article shows their success in two RA patients with perforated corneal ulcer on the articular and the ocular manifestations.

## Case presentation

### Case 1

A 45-year-old Moroccan woman, with the history of thyroidectomy for 18 years ago receiving the thyroid hormone replacement therapy. She had been diagnosed over the previous 17 years with sero-positive erosive and deforming RA, initially treated by Methotrexate given by intramuscular injection at the dose of 20 mg/week (the dose was adjusted to her weight) and 5 mg/day of oral Prednisone. The Methotrexate was stopped for therapeutic inefficacy after one year and substituted by Sulfasalazine at the dose of 2 g/day associated to 10 mg/day of oral Prednisone. The Salazopyrine was also discontinued for inefficacy after two years. In February 2017, the patient was hospitalized for active RA flare with a DAS28 (Disease Activity Score28) at 6.8 when the decision of biotherapy as a treatment was made. She received the first cycle of Rituximab made of two intravenous infusions at 2-week intervals (1 g/infusion) but five months later, she presented another severe RA flare (DAS28 = 6.2) concluding to the clinical and biological failure of Rituximab. The patient remained on oral corticosteroids at 10 mg/day. In July 2017, due to the occurrence of the redness and pain of the left eye with a decrease in the visual acuity, she consulted an ophthalmologist who objectified a perforated corneal ulcer in the left eye and pre-perforated corneal ulcer in the right eye. Concerning her rheumatic disease, she had a RA flare with DAS28 at 5.85. She received in emergency an intravenous high dose of methylprednisolone at the dose of 1 g/day during three consecutive days followed by Infliximab as biologic DMARDs, according to the RA protocol. Infliximab was administrated at 3 mg/kg in first infusion, followed 2 and 6 weeks later by other infusions, then an infusion every 8 weeks. The therapeutic decision was adopted conjointly with her ophthalmologist. Three months after the initiation of the therapy, the RA flare relieved with DAS28 at 3.8 and her eyes improved greatly with complete resolution of the inflammation. This treatment enabled to stabilize the perforated corneal ulcer in the left eye and to stop the progression of pre-perforated corneal ulcer to a veritable perforation. Thirteen months later, the positive ocular and articular therapeutic response was maintained with Infliximab on the follow-up visit with reduced DAS28 at 3.6 leading the ophthalmologist to plan corneal graft.

### Case 2

A 68-year-old Moroccan man with a positive history of smoking habit had been diagnosed with sero-positive erosive and destructive RA and dry-eye syndrome since April 2010. He was treated by Methotrexate given by intramuscular injection at the dose of 20 mg/week (the dose was adjusted to his weight) and 5 mg/day of oral Prednisone. In July 2017, the patient was referred to our hospital by his ophthalmologist for severe RA flare complicated by perforated corneal ulcer of the right eye. The DAS28 was at 6.8. Due to his serious ocular condition complicating an active RA and taking into consideration the successful results of Rituximab as a treatment of ocular manifestations of RA especially PUK described in some articles, we decided, in collaboration with his ophthalmologist, to indicate the Rituximab. The patient received firstly a bolus of methylprednisolone at 500 mg a day during three consecutive days followed by two intravenous infusions of Rituximab at 2-week intervals (1 g/infusion).

Six months later, we observed an interesting improvement with the stabilization of the right eye corneal damage and the resolution of articular symptoms with DAS28 at 3.2. Rituximab therapy was indicated systematically every year with the aim to control the underlying disease and to prepare the eye to corneal graft. Two years after the initiation of the therapy, we noted successful ocular and rheumatic outcomes.

## Discussion and conclusions

In rheumatoid arthritis, ocular involvement is common with an estimated incidence at 25% according to Messmer EM et al. [5] and 39% found in other studies [6, 7]. Among these ocular manifestations, peripheral ulcerative keratitis (PUK) is an inflammatory condition of the peripheral cornea in which corneal meltdown develops in juxta-limbal location. Untreated, it can progress from marginal corneal thinning to perforation, which represents the most severe ocular complication leading to vision loss [1, 8]. The prevalence of PUK is 1 to 3% according to some studies [3, 6]. It occurs in patients with destructive and nodular RA with high titres of rheumatoid factor and anti-cyclic citrullinated peptide anti-bodies [2, 6]. The PUK arises after long years of disease evolution. The studies reported a delay of 17 to 20 years between the diagnosis of RA and the corneal ulcer onset [9–11]. Classically, PUK is manifested by pain and ocular redness, tearing, photophobia or even a decreased visual acuity [9, 12]. The clinical presentation begins with a discrete opacity adjacent to the limbus caused by the stromal cell infiltrate. Subsequently, the ulcer appears and takes the form of a crescent parallel to the limbus with epithelial thinning of the underlying stroma [9, 11]. The PUK is often associated with scleritis or can complicate it particularly the necrotizing type [13].

The pathophysiology of PUK is not fully understood [14, 15]. Many studies identified an abnormal T-cell response by the increased production of helper T-cells that stimulate B-cells, leading to anti-bodies production and the formation of immune complexes. The immune complexes deposed at the periphery of the cornea, activates the classical pathway of the complement system causing a chemoattractant recruitment of inflammatory cells in the peripheral cornea. These cells produce collagenases and other proteases that destroy the corneal stroma [3, 16].

Medical management of this destructive ocular inflammation secondary to RA involves corticosteroids and immunosuppressants. The treatment strategy is based on a therapeutic escalation depending on the severity of the corneal ulcer and the underlying disease [9]. Initially, ophthalmologists should prescribe lubricating agents that help to lubricate the ocular surface and to remove damaging inflammatory proteins and mediators [17]. Local corticosteroids should be avoided because they would reduce collagen synthesis that increase the risk of perforation. Systemic corticosteroids (CS) are the most commonly applied treatment in the management of acute PUK. The usual starting dose is 1 mg/kg/day, but in cases of refractory corneal ulcer with a major risk of vision loss, a high dose of CS are prescribed as a bolus of methylprednisolone at the dose of 1 g/day for three consecutive days followed by oral therapy [13, 18]. According to Foster and Messmer, PUK related to RA should be considered as an indicator of potentially lethal systemic vasculitis that can never be stopped only by local therapies [17, 19]. Therefore, immunosuppressive drugs are proposed in combination with CS in the treatment of this severe eye inflammation [20, 21]. Some studies suggested the use of methotrexate as an initial treatment for most patients, reserving the potentially more toxic agents such as cyclophosphamide and chlorambucil for use in severe progressive cases and in cases unresponsive to methotrexate or other antimetabolites [5, 21, 22]. As reported by Foster and Messmer, cyclophosphamide was the most effective agent for the treatment of PUK in their series [5, 23]. Azathioprine and cyclosporine have been used successfully in some cases [24, 25]. Since the development of biotherapies, small series and clinical cases reported the effectiveness of these drugs as a treatment of ocular conditions complicating the RA. The Infliximab showed a good therapeutic response in the management of these intra ocular inflammatory diseases. Thomas JW et al. demonstrated that Infliximab was effective in three patients with PUK related to RA that was refractory to conventional treatments as it stopped the corneal thinning, reduced the conjunctival inflammation and closed the corneal epithelial defect [26]. Antao SF et al. described the efficacy of Infliximab used at the dose of 5 mg/kg due to the advanced severity of the case by the stabilization of bilateral active corneal melts resistant to all conventional therapies, leading the disease progression to stop after multiple bilateral tectonic keratoplasties [27]. In a few published cases, Infliximab has proved to be the optimal option in refractory cases of RA associated scleritis and PUK [4, 28, 29]. Ashok D et al. reported that the dose at 5 mg/kg of Infliximab was effective in one case of necrotizing scleritis associated to PUK complicating RA [30]. Etanercept has been reported to treat sterile corneal ulceration in inflammatory disease, but it is used with some uncertainty according to some articles [29, 31]. Some cases of PUK related to RA alone or associated with scleritis, have been improved successfully with Rituximab as a treatment [1, 32]. Our cases suggest the efficacy of these biologic agents as a treatment of this severe and destructive keratolysis of the cornea complicating an active RA and exposing the patient to the risk of blindness. This positive therapeutic response obtained by Infliximab and Rituximab contribute to increase literature reports of this therapy success.

The surgical treatment of PUK is indicated in cases of corneal perforation to preserve globe integrity. Different techniques can be carried out depending on the size of ulceration including tissue adhesives, bandage contact lens, lamellar graft, tectonic corneal grafting and amniotic membrane transplantation [5, 11]. The conjunctival resection is a surgical technique that can remove a large number of inflammatory cells, complex immunes and decrease collagenase production, which accelerates the reduction of inflammation [33]. Tissue adhesives are used in patients with important risk of perforation or a perforation less than two mm followed by the application of bandage contact lens [34, 35]. Lamellar or penetrating grafts are recommended in corneal ulceration to prevent perforation when the ulcer is too large for the tissue adhesives [12, 36]. The prognosis of these corneal transplants is often disappointing due to the probability of recurrent tissue destruction in the graft by the initial disease. Therefore, these types of transplants must be effected in patients receiving appropriate immunosuppressive treatment [3, 36].

According to these outcomes, our patients received initially systemic CS to control this serious ocular complication and certainly the severe flare-up of the articular refractory disease. The CS were followed by biological therapy with the aim to obtain quick and effective results in such urgent cases especially after the inefficiency of csDMARDs in the management of the RA. In collaboration with their ophthalmologist and based on prior studies’ findings, we opted for Infliximab in case 1 after the failure of Rituximab as the first proposed biologic agent and Rituximab for case 2. The two biological therapies gave positive results during the follow-up visits after thirteen months (for case 1) and two years (for case 2) from the therapy initiation. They have led to stabilize the perforated corneal ulcers, to arrest progression of pre-perforated corneal ulcer to a veritable perforation and to control the RA as underling systemic vasculitis disease, which will allow their ophthalmologist to schedule keratoplasty.

There is a lack of knowledge concerning the long-term efficacy and safety of the biologic agents for use in ocular inflammation related to systemic disease. Thus, further clinical studies are needed to help guide the treatment of this refractory ocular complication.

## Data Availability

The datasets are available from the corresponding author on reasonable request.

## Abbreviations

CS: Corticosteroids
csDMARDs: Conventional synthetic disease-modifying antirheumatic drugs
DAS28: Disease activity score28
MMP-1: Matrix metalloproteinase-1
PUK: Peripheral ulcerative keratitis
RA: Rheumatoid arthritis
TIMP-1: Tissue inhibitor of metalloproteinase-1

## References

[1] Hardy S, Hashemi K, Catanese M, Candil M, Zufferey P, Gabison E, Guex-Crosier Y (2017). Necrotising Scleritis and peripheral ulcerative keratitis associated with rheumatoid arthritis treated with rituximab. Klin Monbl Augenheilkd.

[2] Watanabe R, Ishii T, Yoshida M, Takada N, Yokokura S, Shirota Y, Fujii H, Harigae H (2017). Ulcerative keratitis in patients with rheumatoid arthritis in the modern biologic era: a series of eight cases and literature review. Int J Rheum Dis.

[3] Artifoni M, Rothschild PR, Brézin A, Guillevin L, Puéchal X (2014). Ocular inflammatory diseases associated with rheumatoid arthritis. Nat Rev Rheumatol.

[4] Atchia II, Kidd CE, Bell RW (2006). Rheumatoid arthritis-associated necrotizing scleritis and peripheral ulcerative keratitis treated successfully with infliximab. J Clin Rheumatol.

[5] Messmer EM, Foster CS (1995). Destructive corneal and scleral disease associated with rheumatoid arthritis. Medical and surgical management. Cornea.

[6] Vignesh AP, Srinivasan R (2015). Ocular manifestations of rheumatoid arthritis and their correlation with anti-cyclic citrullinated peptide antibodies. Clin Ophthalmol.

[7] Reddy SC, Gupta SD, Jain IS, Deodhar SD (1977). Ocular manifestations of rheumatoid arthritis. Indian J Ophthalmol.

[8] Almaliotis D, Zakalka M, Gerofotis A, Chatzicharalampous K, Efstathiou M, Daniilidis M (2016). Ocular manifestations in rheumatoid arthritis. Open J Opthalmol.

[9] Gueudry J, Muraine M (2015). Ulcères et ulcérations cornéennes chroniques. Rapport de la Société Française d’ophtalmologie.

[10] McKibbin M, Isaacs JD, Morrell AJ (1999). Incidence of corneal melting in association with systemic disease in the Yorkshire region. Br J Ophthalmol.

[11] Galor A, Thorne JE (2007). Scleritis and peripheral ulcerative keratitis. Rheum Dis Clin.

[12] Hick S, Duchesne B, Kaye O, Maréchal-Courtois C, Galand A (2002). Les ulcères de cornée associés à la polyarthrite rhumatoïde. Rev Med Liege.

[13] Hata M, Nakamura T, Sotozono C, Kumagai K, Kinoshita S, Kurimoto Y (2012). A typical continuous keratitis in a case of rheumatoid arthritis accompanying severe scleritis. Cornea.

[14] Tlucek PS, Stone DU (2012). Certolizumab pegol therapy for rheumatoid arthritis-associated scleritis. Cornea.

[15] Smith VA, Rishmawi H, Hussein H, Easty DL (2001). Tear film MMP accumulation and corneal disease. Br J Ophthalmol.

[16] John SL, Morgan K, Tullo AB, Holt PJ (1992). Corneal autoimmunity in patients with peripheral ulcerative keratitis (PUK) in association with rheumatoid arthritis and Wegener's granulomatosis. Eye (Lond).

[17] Messmer EM, Foster CS (1999). Vasculitic peripheral ulcerative keratitis. Surg Ophthalmol.

[18] Chow CY, Foster CS (1996). Mooren’s ulcer. Int Ophthalmol Clin.

[19] Foster CS, Forstot SL, Wilson LA (1984). Mortality rate in rheumatoid arthritis patients developing necrotizing scleritis or peripheral ulcerative keratitis: effects of systemic immunosuppression. Ophthalmology.

[20] Sato T, Minakuchi S, Mochizuki M, Takeuchi M (2014). Acute anterior uveitis after discontinuation of tocilizumab in a patient with rheumatoid arthritis. Clin Ophthalmol.

[21] Squirrell DM, Winfield J, Amos RS (1999). Peripheral ulcerative keratitis “corneal melt” and rheumatoid arthritis: a case series. Rheumatology.

[22] Jabs DA, Rosenbaum JT, Foster CS, Holland GN, Jaffe GJ, Louie JS (2000). Guidelines for the use of immunosuppressive drugs in patients with ocular inflammatory disorders: recommendations of an expert panel. Am J Ophthalmol.

[23] Stylianides A, Jones MN, Stewart RM, Murphy CC, Goodson NJ, Kaye SB (2013). Rheumatoid arthritis-associated corneal ulceration: mortality and graft survival. Ophthalmology.

[24] Galor A, Jabs DA, Leder HA, Kedhar SR, Dunn JP, Peters GB (2008). Comparison of antimetabolite drugs as corticosteroid-sparing therapy for noninfectious ocular inflammation. Ophthalmology.

[25] McCarthy JM, Dubord PJ, Chalmers A, Kassen BO, Rangno KK (1992). Cyclosporine a for the treatment of necrotizing scleritis and corneal melting in patients with rheumatoid arthritis. J Rheumatol.

[26] Thomas JW, Pflugfelder SC (2005). Therapy of progressive rheumatoid arthritis-associated corneal ulceration with infliximab. Cornea.

[27] Antao SF, Ayoub T, Tahir H, Parmar DN (2012). Stabilization of bilateral progressive rheumatoid corneal melt with infliximab. Case Rep Ophthalmol Med.

[28] Herrera-Esparza R, Avalos-Díaz E (2009). Infliximab treatment in a case of rheumatoid scleromalacia perforans. Reumatismo..

[29] Sassa Y, Kawano Y, Yamana T, Mashima T, Ishibashi T (2012). A change in treatment from etanercept to infliximab was effective to control scleritis in a patient with rheumatoid arthritis. Acta Ophthalmol.

[30] Ashok D, Ayliffe WH, Kiely PDW (2005). Necrotizing scleritis associated with rheumatoid arthritis: long-term remission with high-dose infliximab therapy. Rheumatology (Oxford).

[31] Smith JR, Levinson RD, Holland GN, Jabs DA, Robinson MR, Whitcup SM (2001). Differential efficacy of tumor necrosis factor inhibition in the management of inflammatory eye disease and associated rheumatic disease. Arthritis Rheum.

[32] Freidlin J, Wong IG, Acharya N (2007). Rituximab treatment for peripheral ulcerative keratitis associated with Wegener's granulomatosis. Br J Ophthalmol.

[33] Feder RS, Krachmer JH (1984). Conjonctival resection for treatment of the rheumatoid corneal ulceration. Ophthalmology.

[34] Vera L, Benzerroug M, Gueudry J, Varin R, Haghighat S, Gerard G (2009). Mise au point sur l’utilisation des colles tissulaires en ophtalmologie. J Fr Ophtalmol.

[35] Gregory JK, Foster CS (1996). Peripheral ulcerative keratitis in the collagen vascular diseases. Int Ophthalmol Clin.

[36] Maeno A, Naor J, Lee HM, Hunter WS, Rootman DS (2000). Three decades of corneal transplantation: indications and patient characteristics. Cornea.

